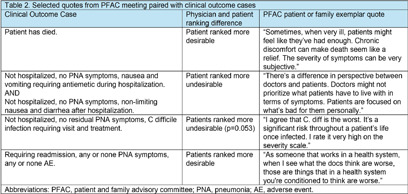# Developing a Desirability of Outcome Ranking for Adults with Non-severe Community-acquired Pneumonia: physician and patient preferences

**DOI:** 10.1017/ash.2025.264

**Published:** 2025-09-24

**Authors:** Eli Finer, Michael Pulia, James Harrison, Jessica Howard-Anderson, Andrea White, Stephanie Edwards, Austin Smith, Payal Patel, Joseph Bledsoe, Jason Carr, Troy Madsen, Valerie Vaughn

**Affiliations:** 1 University of Utah Hospital; 2 University of Wisconsin-Madison; 3 Division of Hospital Medicine, University of California San Francisco; 4 Emory University School of Medicine; 5 University of Utah School of Medicine; 6 University of Utah; 7 Intermountain Healthcare; 8 Harris Carmichael, Intermountain Health; 9 Intermountain Medical Center; 10 Park City Hospital; 11 University of Utah School of Medicine

## Abstract

**Background:** Dichotomous outcomes rarely capture the range of potential outcomes important to patients and clinicians. To address this limitation, the Desirability of Outcome Ranking (DOOR) score was created to rank potential outcomes from least to most desirable. Currently, there is no standardized method to develop a DOOR score and data are limited on whether patients and their clinicians rank outcomes similarly. We aimed: (a) to develop a novel DOOR score for adults hospitalized with community-acquired pneumonia (CAP) by surveying patients and clinicians on their preferred outcome ranking and (b) to compare their relative DOOR rankings. **Methods:** We created nine clinical scenarios describing the spectrum of potential outcomes of patients with CAP two weeks after initial emergency department visit. To ascertain clinician DOOR score, we used a snowball sampling method to recruit a target of 25 clinicians in specialties that regularly treat CAP. For the patient DOOR score, we recruited patients hospitalized with CAP by reviewing electronic patient lists for adults hospitalized with pneumonia. Respondents were asked to rank the 9 cases from most to least desirable in REDCap. To create the final DOOR score, we used Friedman rank sum tests to combine/collapse DOOR outcomes with scores that did not significantly differ. We used the Mann Whitney U test to compare DOOR rankings between physicians and patients. Final study results were presented to a national hospital medicine patient and family advisory committee (PFAC) for their impressions. **Results:** 22 patients (71% response rate) and 25 clinicians responded to our DOOR survey. Their ranked order of DOOR outcomes is shown in Table 1. Combining non-significantly different DOOR outcomes resulted in collapsing of 6 cases into 2 categories for 5 overall DOOR scores that significantly differed from each other (Table 1 for final ranking). Patients and clinicians had significantly different preferred ranking for 6 DOOR cases. Our PFAC had several hypotheses as to why rankings differed (Table 2). **Conclusion:** We present a novel DOOR score derived from patient and clinician reported preferences for outcomes of hospitalized adult patients with CAP. Clinicians and patients differed in their perception of certain outcomes with patients ranking symptoms that were uncomfortable but not potentially life-threatening as less desirable than physicians. Physicians tended to rank quality linked metrics such as readmission as worse than patients. When designing future trials using DOOR scores, researchers should consider including patients in DOOR score design as their perspectives may differ from clinicians.

**Figure:**
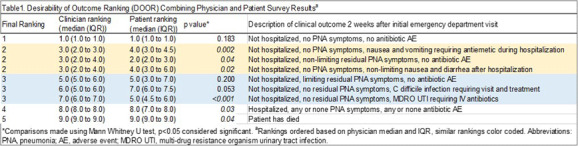

**Figure:**